# Encephalitis and aseptic meningitis: short-term and long-term outcome, quality of life and neuropsychological functioning

**DOI:** 10.1038/s41598-019-52570-2

**Published:** 2019-11-06

**Authors:** Else Quist-Paulsen, Vidar Ormaasen, Anne-Marte B. Kran, Oona Dunlop, Per Magne Ueland, Thor Ueland, Randi Eikeland, Pål Aukrust, Tonje H. Nordenmark

**Affiliations:** 1 Department of Infectious Diseases, Oslo University Hospital Ullevål, Oslo, Norway; 2 Institute of Clinical Medicine, University of Oslo, Oslo, Norway; 3 Faculty of Medicine, University of Oslo, Oslo, Norway; 4 Department of Microbiology, Oslo University Hospital Ullevål, Oslo, Norway; 5 Department of Acute Medicine, Oslo University Hospital Ullevål, Oslo, Norway; 6 Bevital A/S, Bergen, Norway; 7 Research Institute of Internal Medicine, Oslo University Hospital Rikshospitalet, Oslo, Norway; 8 K.G. Jebsen - Thrombosis Research and Expertise Center (TREC), University of Tromsø, Tromsø, Norway; 9 Sørlandet Hospital, National Advisory Unit on Tick-borne Diseases, Kristiansand, Norway; 10 Section of Clinical Immunology and Infectious Diseases, Oslo University Hospital Rikshospitalet, Oslo, Norway; 11 Department of Physical Medicine and Rehabilitation, Oslo University Hospital Ullevål, Oslo, Norway

**Keywords:** Meningitis, Central nervous system infections

## Abstract

For those surviving encephalitis, the influence on daily life of patients and their relatives may be substantial. In contrast, the prognosis after aseptic meningitis (ASM) is considered good. In this prospective study in patients with encephalitis (n = 20) and ASM (n = 46), we show that both groups experienced reduced Health Related Quality of Life (HRQoL) at two months after discharge, and that workability was reduced in 37% of the patients with ASM. However, 12 months after discharge no neuropsychological deficits were detected in the ASM group, whereas patients with encephalitis had lower scores on tests of fine motor and psychomotor skills as well as on learning and memory. We also found that for patients with encephalitis, neopterin, as a marker of Th1 cell induced macrophage activation, and a putatively neurotoxic ratio of the kynurenine pathway (KP) measured during the acute phase was associated with lower HRQoL. Our data show that not only encephalitis, but also ASM has substantial short-term influence on HRQoL and workability. For patients with encephalitis we suggest a link between immune activation and activation of the KP during the acute phase with impaired HRQoL.

## Introduction

Infectious encephalitis is an inflammatory condition of the brain parenchyma. The most common identified cause is the herpes simplex type I (HSV-1), which left untreated has a mortality of 70%^1^. In contrast, aseptic meningitis (ASM) is considered a more benign condition with low mortality even without specific treatment^2^.

Studies regarding quality of life and neurocognitive sequela after encephalitis and ASM are scarce, most studies are agent specific (e.g. only HSV-1) and comparison between studies is hampered by divergent inclusion criteria, diversity in tests performed and follow-up time^3–5^. Many patients with encephalitis remain undiagnosed regarding causing agent, and few studies have investigated outcome for various or unknown etiologies^6,7^. Data concerning outcome and disability after ASM vary from no complains, to reduced Health Related Quality of Life (HRQoL), fatigue, and reduced cognitive function^8–12^.

Except from being vital for pathogen clearance in the CNS, several studies have demonstrated that immune activation and activation of the kynurenine pathway (KP) is associated with outcome of CNS infections^13–17^. Whereas a balanced immune response is beneficial for the host, an overwhelming activation of inflammatory pathways could be harmful. Activation of the KP pathway can also mediate harmful as well as protective effect on CNS during infections, depending on the balance between the metabolites of KP. Kynurenic acid (KYNA) has neuroprotective effects whereas quinolinic acid (QA) mediates excitotoxicity, and both have been associated with depression and impaired cognitive function^18–20^. Recently we reported a state of generalized immune activation and increased levels of KP metabolites in the cerebrospinal fluid (CSF) from patients with encephalitis and ASM compared to patients without CNS infection^21^. Moreover, for patients with encephalitis we found that neopterin, as a marker of interferon (IFN)-γ activity, correlated with the rate-limiting step of the KP (i.e. indoleamine 2,3-dioxygenase (IDO)) and with a putative neurotoxic ratio of the KP.

In the present study, we aimed to evaluate workability and HRQoL in patients with acute encephalitis and ASM of various and unknown etiology two months after discharge. Secondly, we accessed long-term neuropsychological outcome at 12 months. We also aimed to investigate whether short-term outcome was related to clinical findings and previously reported, dysregulated neopterin and KP metabolites during the acute phase of the infection.

## Methods

### Study design and patients

**Figure 1:**
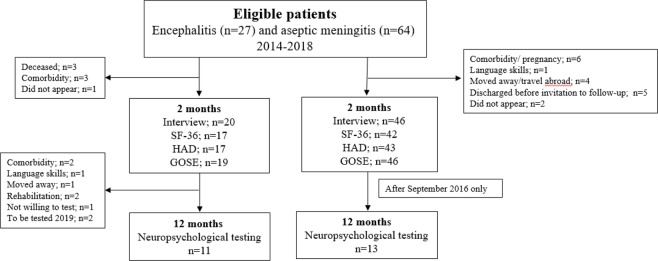
Flowchart showing inclusion of patients. SF-36: Survey Short Form, HAD: Hospital Anxiety and Depression Scale, GOSE: Glasgow Outcome Scale Extended.

This prospective observational study was performed at Oslo University Hospital, Ullevål between January 2014 and June 2018. During the first two years (2014–2015) all patients presenting with acute symptoms of CNS infection at the Department of Medicine and Neurology who underwent a lumbar puncture (LP) were included (n = 244). Of these, 32 of the 45 patients who fulfilled the case definition of ASM and 12 of the 19 patients who fulfilled the inclusion criteria of encephalitis were included in the follow-up study. During the last inclusion period (March 2016-June 2018) only patients from the Department of Infectious Diseases were eligible for inclusion, of 19 cases with ASM, 14 patients were included, whereas all patients diagnosed with encephalitis in this latter inclusion period fulfilled inclusion criteria for the follow-up study (Fig. 1). The case definition of encephalitis is, as previously published, based on the same criteria and symptoms as stated in the International Encephalitis Consortium case definition (Table 1)^22,23^. The case definition of ASM is based on a consortium definition published by Tapiainen *et al*. (Table 1)^24^. Nine patients with ASM had prior to the lumbar puncture been treated with antibiotics, which could results in false negative CSF culturing. For three of these patients, no causing agent was identified, and a negative polymerase chain reaction (PCR) for common causes of bacterial meningitis was required to fulfill the case definition. None of the patients had a positive blood culture. For all patients in this study, in-house real time polymerase chain reaction (PCR) for detection of herpes simplex virus 1 (HSV1) and 2 (HSV2), varicella zoster virus (VZV) and enterovirus were carried out. Analyses for detection of other microbiological agents in CSF and serum were analyzed if clinically relevant^23^.

**Table 1:** Case definitions.

Condition	Case definition
Encephalitis	Encephalopathy (altered mental function or change in personality) for >24 hours with no other cause identified and at least two of the following:
	1. ≥ 5 × 10^6^/L leucocytes (WBC) in cerebrospinal fluid (CSF)
	2. new onset of seizures,
	3. new onset of focal neurology,
	4. documented fever >38°C before or within 24 hours after hospitalisation,
	5. EEG findings suggestive of encephalitis and/or
	6. MRI/CT findings suggestive of encephalitis
Aseptic meningitis (ASM)	1. Clinical signs of meningitis (headache, neck stiffness, photophobia and/or fever),
	2. ≥ 5 × 10^6^/L leucocytes in CSF and
	3. culture and microscopy negative CSF^a^

^a^For patients treated with antibiotics prior to LP, a negative bacterial PCR for common causes of bacterial meningitis was required.

A follow up appointment was scheduled two months after discharge (Fig. 1). Patients with encephalitis and patients diagnosed with ASM after September 2016 were invited to neuropsychological testing 12 months after infection. Patients with premorbid chronic psychiatric disease, addictive disorders or patients with poor Norwegian language skills were excluded. Two patients in the encephalitis group had recently been tested and were excluded from the neuropsychological testing due to test-retest bias (Fig. 1).

### Patient characteristics and clinical data

Clinical and demographic data were obtained during hospital admittance as described in our previous work (Table 2)^23^. Etiological agents were classified using the criteria given in the review of Granerod *et al*.^25^. From seven patients with encephalitis and 10 ASM patients, measurements of tryptophan [TRP], kynurenine [KYN], anthranilic acid [AA], kynurenic acid [KYNA], 3-hydroxykynurenine [3-HK], 3-hydroxyanthranilic acid [3-HAA], quinolinic acid [QA], picolinic acid [PIC], and neopterin from CSF and serum sampled at admission were known. These measurements were analyzed by liquid chromatography- tandem mass spectrometry (LC-MS/MS) as previously described^21,26^. From previous published data, the calculated ratio (KYN/TRP) of the rate-limiting enzyme of the KP in the CNS, i.e. indolamine 2,3-dioxygenase (IDO) and the putatively neuroprotective (i.e. KYNA) to neurotoxic KP metabolites (i.e. 3-HK + QA) were known^21^.

### Short-term follow up

The clinical follow-up included a structured interview on persisting complains and workability (Table 3), the Glasgow Outcome Scale extended (GOSE)^27^, the Hospital Anxiety and Depression Scale (HAD)^28^ and the Survey Short Form (SF-36)^29^. The eight scales of the SF-36 were aggregated in two summary component scores, the Physical (PCS) and Mental (MCS) Component Summary scores^30^.

**Table 2:** Demographic, laboratory and clinical characteristics of patients.

	Encephalitis (n = 20)	Aseptic meningitis (n = 46)	p-value
Gender, male, no (%)	10/20 (50)	16/46 (35)	ns
Age^b^	53 (29)	34 (11)	<0.001
Stay in intensive care unit (%)	8/20 (40)	2/46 (4)	<0.001
Etiology, confirmed/probable^c^ (%)	5/20 (25)	34/46 (74)	<0.001
Days in hospital^b^	14 (13)	3 (2)	<0.001
Days since ictus^b^	1 (3)	1 (1)	ns
Headache (%)	12/20 (60)	46/46 (100)	<0.001
Neck stiffness, subjective (%)	3/19 (16)	31/46 (67)	<0.001
Photophobia (%)	7/20 (35)	32/46 (70)	0.009
**Clinical findings**			
Objective neck stiffness (MD) (%)	5/19 (26)	22/46 (48)	ns
Seizures (%)	2/17 (12)	0	0.027
Focal findings (%)	9/20 (45)	3/ 42 (7)	<0.001
Objective fever (%)	14/20 (70)	29/46 (63)	ns
Temperature, admission^a^	37.3 (1.0)	37.3 (0.9)	ns
MR typical for encephalitis (n = 31)	4/18 (22)	0/13 (0)	ns
EEG suggestive of encephalitis (n = 18)	10/16(63)	0/2 (0)	ns
**Laboratory findings**			
WBC in serum (n = 66)^a^	9.7 (3.1)	8.9 (2,3)	ns
CRP (n = 65)^b^	5.7 (21)	4.7 (9)	ns
CSF- WBC^b^	65 (256)	243 (409)	ns
CSF- proteins^b^	0.909 (0.907)	0.675 (0.531)	0.017
CSF- glucose^a^	3.5 (0.6)	3.3 (0.6)	ns
CSF- glucose ratio (n = 50)^a^	0.56 (0.13)	0.56 (0.09)	ns
**Treatment during hospital stay**			
Aciclovir iv, no (%)	18/20 (90)	34/46 (74)	ns
“CNS- antibiotics”, no (%)	14/20 (70)	28/46 (61)	ns

Data are presented as number (%), ^a^mean (SD) or ^b^median (IQR). ^c^Etiology in encephalitis were; VZV (n = 2), HSV1 (n = 1), *B. burgdorferi* (n = 2). Aseptic meningitis; EV (n = 20), HSV2 (n = 10), VZV (n = 2), Toscana virus (n = 1), *B. burgdorferi* (n = 1).

### Neuropsychological testing and long-term follow up

A wide range of neuropsychological tests was used to measure cognitive function 12 months after discharge from hospital. The chosen neuropsychological test battery was similar to that used in a follow-up study on patients with neuroborreliosis^31^, and was designed to evaluate a broad range of cognitive abilities (Table 4)^32–39^.

**Table 3:** Short- term outcomes (at 2 months).

	Encephalitis (n = 20)	Aseptic meningitis (n = 46)	p-value
Time to follow-up, days^b^	79 (60)	62 (44)	0.048
No reported complaints (%)	1/20 (5)	17/46 (37)	0.007
Sick leave, total no (%)	12/12 (100)	16/43 (37)	<0.001
100% unable to work (%)	7/12 (58)	5/43 (12)	0.002
**GOSE**			
GOSE <4	1 (5)	0	0.007
GOSE = 5–6	14 (74)	18 (39)	
GOSE = 7–8	4 (21)	28 (61)	
**SF-36 subscores** ^**a**^			
Physical functioning (PF)	78.5 (21)	89.3 (18)	ns
Role physical (RP)	16.2 (32)	45.2 (48)	0.01
Role emotional (RE)	58.8 (42)	77.4 (38)	ns
Bodily pain (BP)	68.9 (28)	65.7 (25)	ns
General health (GH)	61.8 (21)	66.6 (27)	ns
Vitality (VT)	45 (15)	51.1 (23)	ns
Social functioning (SF)	61.8 (26)	71.1 (31)	ns
Mental health (MH)	73.2 (14)	79.2 (15)	ns
Physical component summary (PCS)	43.6 (8)	46.7 (10)	ns
Mental component summary (MCS)	45 (8)	49 (11)	ns
**HAD**			
HAD sum A, median			
Sum 0–7, no of patients (%)	15 (88)	34 (79)	ns
Sum 8–10, no of patients (%)	1 (6)	5 (12)	ns
Sum 11–21, no of patients (%)	1 (6)	4 (9)	ns
HAD, sum D, median			ns
Sum 0–7, no of patients (%)	16 (94)	40 (93)	ns
Sum 8–10, no of patients (%)	1 (6)	2 (5)	ns
Sum 11–21, no of patients (%)	0	1(2)	ns
**Reported subjective complaints**			
Headache (%)	6/20 (30)	23/46 (50)	ns
Neck stiffness (%)	0	4/44 (9)	ns
Photophobia (%)	7/18 (39)	8/46 (17)	ns
Phonophobia (%)	7/18 (39)	12/46 (26)	ns
Neurological symptoms^c^ (%)	13/20 (65)	11/46 (24)	0.001
Concentration difficulties (%)	12/20 (60)	13/46 (28)	0.015
Memory problems (%)	14 /20 (70)	17/46 (37)	0.013
Emotional change^d^ (%)	7/18 (39)	13/46 (28)	ns
Extensive tiredness (%)	15/20 (75)	18/46 (39)	0.007
Sleep disturbance^e^ (%)	14/20 (70)	14/46 (30)	0.003

Data are presented as number of patients (%), ^a^mean (SD) or ^b^median (IQR). ^c^Most reported were difficulty finding words, reduced coordination, urinary retention. ^d^Feeling of anxiousness or mood disorder, ^e^increased need of sleep. GOSE: Glasgow Outcome Scale Extended, SF-36: Survey Short Form, HAD: Hospital Anxiety and Depression Scale.

### Ethical considerations

All patients gave a written informed consent. The study was approved by The Regional Committees for Medical and Health Research Ethics (REC South East, reference number 2011/2578) and the hospital ethical council, and was conducted according to relevant guidelines and regulations.

### Statistics

Categorical variables are expressed as counts (percentages) and comparisons between groups were done using Pearson Chi-square test for categorical data. Continuous data are presented as mean (SD) if normally distributed, otherwise as median (interquartile range, IQR). Comparisons of continuous data were analyzed using T-test for normally distributed data, otherwise Mann-Whitney-U was used. Results of questionnaires and neuropsychological tests are presented as mean raw scores with standard deviations (SD). One-sided T-test was used to compare SF-36 data to normative data matched on age and sex^40,41^. For PCS and MCS a population mean of 50 was used. To compare results of neurocognitive tests with expected mean, z-scores were calculated. A z-score below −1 SD was considered a deficit. Associations between HRQoL and measurements of immune activation and KP metabolites at admittance were performed with Spearman’s rank correlation. Due to the limited sample size, we restricted our correlation analysis with SF-36 measures to previously identified dysregulated KP pathway measures (i.e. neopterin, IDO and KYNA/(3-HK + QA)) to minimize the influence of multiple testing^30^. To limit type II statistical errors, no correction for multiple comparisons was made in this explorative study^42^. All data analyses were performed in SPSS version 24 (IBM Corp. Armonk, NY, USA) and graphs generated by Graphad Prism 8 (GraphPad, San Diego, USA).

## Results

### Patient characteristics prior to discharge

Three patients (11%) with encephalitis died during the hospital stay. Etiology was identified in only 5/20 surviving patients with encephalitis. For patients with ASM, the causing agent was identified in 34/ 46 (74%). Enterovirus was the most common cause, identified in 43% of patients with ASM. Interestingly, we identified *Borrelia burgdorferi* as causing agent in two patients with encephalitis, as well as in one patient with aseptic meningitis. Clinical characteristics, findings and treatment of patients at admission are shown in Table 2.

### Short-term outcome

**Figure 2:**
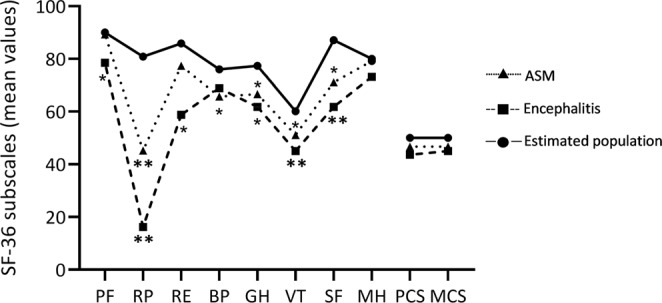
Mean scores of SF-36 subscales, PCS and MCS of patients with aseptic meningitis and encephalitis compared to the estimated population mean at follow-up at two months. Asterisks denotes significant difference vs estimated population mean; *p < 0.05, **p ≤ 0.001. For encephalitis both PCS (p = 0.005) and MCS (p = 0.014) were below the population mean, while for ASM, only PCS was below (p = 0.042). PF: Physical functioning, RP: Role physical, RE: Role emotional, BP: Bodily pain, GH: General health, VT: Vitality, SF: Social functioning, MH: Mental health, PCS: Physical Component Summary score, MCS: Mental Component Summary score.

At a median of 67 days (range 36–168), all employed patients with encephalitis and 37% of the ASM patients had reduced workability. Moderate disability, i.e. GOSE ≤ 6, was found in 39% of patients with ASM, and in 75% of patients with encephalitis (Table 3). Of patients with ASM, 63% reported daily symptoms, while all but one patient (95%) with encephalitis experienced symptoms (Table 3). In general, scores of the SF-36 subscales and the component summary score (PCS and MCS) were lower for patients with encephalitis compared to ASM patients (Table 3). For encephalitis, both PCS and MCS were significantly lower compared to the expected population mean of 50 (PCS 43.6, p = 0.005 and MCS 44.9, p = 0.014) (Fig. 2). For ASM, only PCS was significantly lower compared to population mean (PCS 46.7, p = 0.042).

### Long-term outcome and neurocognitive function

The education level was not different between the groups, but the education level was high, 15.5 ± 3 yrs. Moreover, tested encephalitis patients were older (53.5 ± 16.5 vs 37.2 ± 9.7, p = 0.007) and more often men (8/11 (72%) vs 3/13 (23%), p = 0.014). The neuropsychological tests were poorer in the encephalitis group regarding fine motor and psychomotor skills, as well as learning and memory (Table 4). The total number of patients with z-scores below −1 SD was 14% in the encephalitis group compared to 3% in the ASM group.

**Table 4:** Neuropsychological test results, SCL-90 and BRIEF (12 months, raw scores).

		Encephalitis (n=11) Mean (SD)	Meningitis (n=13) Mean (SD)	p-value
**Neuropsychological test battery**				
Attention	Ruff 2&7 Total Accuracy^a^	94.9 ± 13.5	102.9 ± 8.6	ns
	Digit span Total^a^	24.0 ± 8.8	28.7 ± 6.1	ns
Psychomotor speed	Ruff 2&7 Total Speed^a^	84.6 ± 33.1	94.9 ± 19.2	ns
	Trail Making test 1^b^	24.6 ± 8.3	17.8 ± 6.4	ns
	Trail making test 2^b^	41.6 ± 28.2	27.4 ± 7.3	0.008
	Trail making test 3^b^	43.4 ± 37.0	28.3 ± 9.0	0.019
	Color word interference test 1^b^	34.7 ± 8.4	27.3 ± 3.5	0.010
	Color word interference test 2^b^	24.6 ± 4.6	20.8 ± 4.6	ns
	Digit symbol^a^	57.5 ± 16.8	72.6 ± 13.2	ns
Fine motor speed	Pegboard Dominant Hand^b^	76.5 ± 18.3	60.0 ± 5.2	<0.001
	Pegboard Non-dominant Hand^b^	83.6 ± 25.1	67.1 ± 8.5	0.028
Verbal learning and memory	CVLT Total learning^a^	53.6 ± 11.3	64.9 ± 7.4	0.040
	CVLT short term memory^a^	11.0 ± 4.0	14.4 ± 2.1	0.005
	CVLT long term memory^a^	11.9 ± 3.1	15.0 ± 1.5	0.008
	CVLT recognition^a^	15.2 ± 1.1	15.7 ± 0.6	0.038
	CVLT false positive^a^	1.6 ± 3.9	0.8 ± 0.3	0.022
Visual learning and memory	BVMT Total learning^a^	23.9 ± 8.1	27.9 ± 4.7	0.049
	BVMT long term memory ^a^	9.6 ± 2.2	10.5 ± 1.3	ns
Vocabulary (total correct)	Vocabulary^a^	31.3 ± 12.0	40.0 ± 7.9	ns
Visuospatial function	Block design^a^	41.7 ± 13.7	45.9 ± 9.8	ns
Executive function	Trail making test 4^b^	52.1 ± 16.5	77.1 ± 31.1	ns
	Color word interference test 3^b^	70.6 ± 18.1	49.5 ± 2.9	0.011
	Color word interference test 4^b^	71.5 ± 15.7	58.4 ± 11.0	ns
	Word Fluency test FAS^a^	42.4 ± 14.5	51.0 ± 14.5	ns
**Symptom burden**				
SCL-90^a^	SCL-90 somatization	8.5 ± 12.1	5.2 ± 6.8	ns
	SCL-90 obsessive compulsive	5.7 ± 4.9	5.0 ± 5.7	ns
	SCL-90 interpersonal sensitivity	1.8 ± 2.2	1.9 ± 2.1	ns
	SCL-90 depression	3.6 ± 4.7	5.4 ± 7.4	ns
	SCL-90 anxiety	1.9 ± 3.0	3.1 ± 3.8	ns
	SCL-90 hostility	0.6 ± 0.8	1.7 ± 2.2	0.021
	SCL-90 phobia	0.5 ± 0.9	0.5 ± 1.0	ns
	SCL-90 paranoid anxiety	0.4 ± 0.5	0.8 ± 1.1	ns
	SCL-90 psychoticism	0.4 ± 0.7	1.1 ± 1.4	ns
	SCL-90 GSI	26.5 ± 28.0	28.0 ± 8.3	ns
BRIEF^a^	BRIEF-A Behavioural Regulation Index	36.7 ± 5.9	36.3 ± 5.1	ns
	BRIEF-A Metacognition Index BRIEF-A Global Executive	50.4 ± 10.4 87.1 ± 15.8	50.6 ± 10.9 83.1 ± 13.8	ns ns

Raw scores of test given as ^a^points, and ^b^seconds CVLT: Californian Verbal Learning Test, BVMT: Brief Visuospatial Memory Test, BRIEF: Behavior Rating Inventory of Executive Function, SCL: Symptom Checklist 90.

### Association of short-term outcome with KP metabolites and markers of inflammation in CSF at admittance

**Figure 3:**
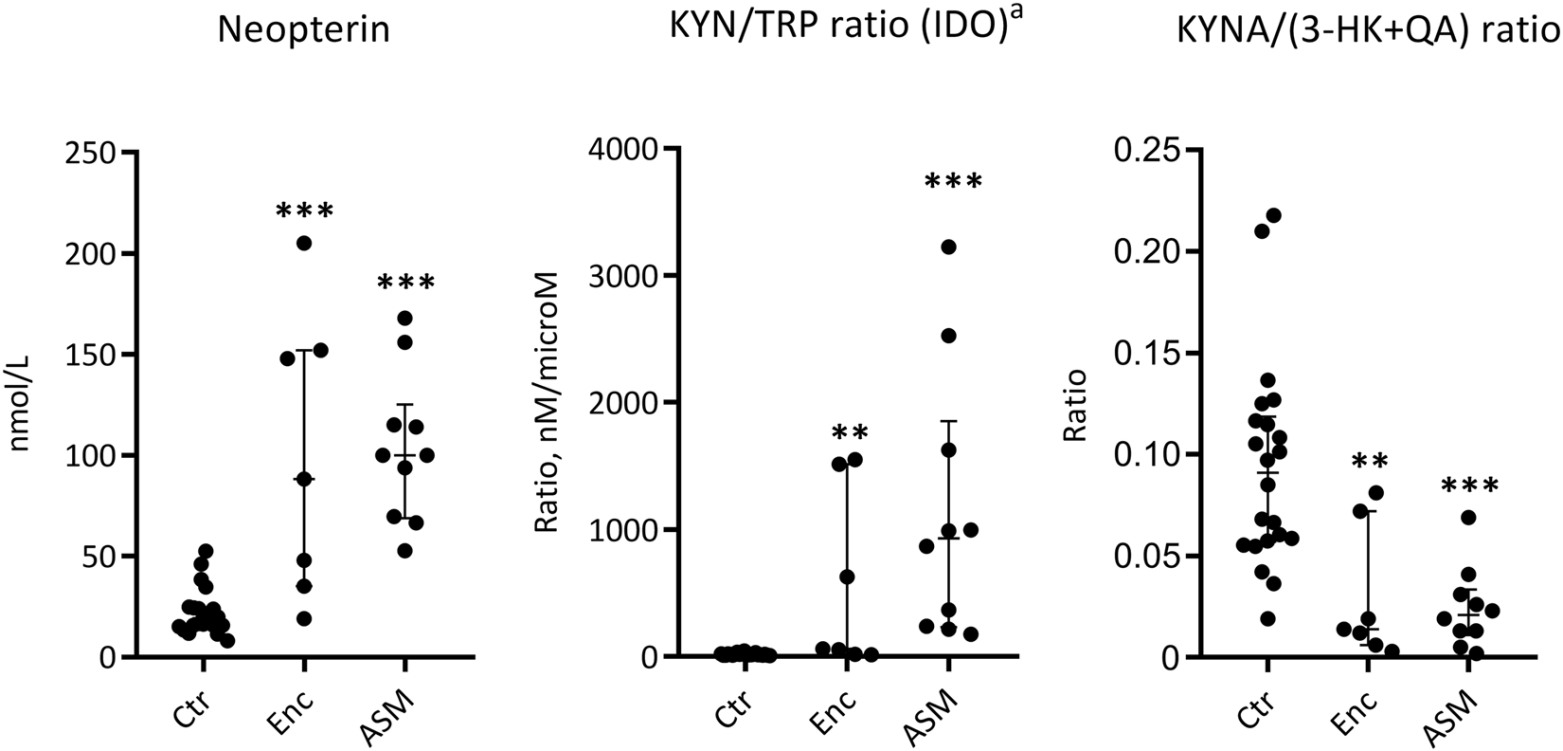
Neopterin, KYN/TRP ratio and KYNA/(3-HK + QA) for patients with encephalitis (n = 7) and ASM (n = 10) measured at admission in comparison with a previously reported control group consisting of patients with no pleocytosis in the CSF^21^. Data shown are medians with IQR. Asterisks above patients groups indicate significant difference vs controls (Mann Whitney U test); **p < 0.01, ***p < 0.001. ^a^KYN/TRP ratio as a measure of IDO activity. KYN: kynurenine, TRP: tryptophan, KYNA: kynurenic acid, 3-HK: 3-hydroxykynurenine, QA: quinolinic acid.

We have previously reported markedly enhanced neopterin and IDO levels and a lower ratio of KYNA/(3-HK + QA), particularly in patients with encephalitis, compared to healthy controls^21^. These parameters in the current sub-population are shown in Fig. 3. To evaluate whether outcome could be related to level of immune activation or activation of the KP, the summary scores of the SF-36 (i.e. PCS and MCS) were used. In patients with encephalitis there was a strong positive correlation between MCS and the putative neuroprotective/neurotoxic ratio of KYNA/ (3-HK + QA) (Rho 0.9, p = 0.014) and strong inverse correlation with neopterin, as a marker of Th1 cell activation (Rho −0.9, p = 0.007), and IDO (Rho −0.9, p = 0.014) (Table 5). No correlation was found between number of days admitted, CSF white cell counts (WBC) or CSF protein at hospital admittance and PCS and MCS.

**Table 5:** Correlations of PCS and MCS at two months with KP metabolites and neopterin in CSF

	Encephalitis (n = 7)		Aseptic meningitis (n = 10)	
	PCS	MCS	PCS	MCS
KYNA/(3-HK + QA)	−0.4 (p = 0.9)	0.9 (p = 0.014)	0.1 (p = 0.7)	−0.5 (p = 0.1)
Neopterin	0.07 (p = 0.9)	−0.9 (p = 0.007)	−0.2 (p = 0.7)	0.09 (p = 0.8)
IDO	0.143 (p = 0.8)	−0.9 (p = 0.014)	−0.2 (p = 0.8)	0.006 (p = 0.9)

Data shown are obtained by Spearman’s rank correlation (p-value). KP: Kynurenine pathway, KYNA: Kynurenic acid, 3-HK: 3-hydroxykynurenine, QA: Quinolinic acid, IDO: KYN (nmol)/TRP(μmol), PCS: Physical Component Summary score, MCS: Mental Component Summary score.

## Discussion

We evaluated short- and long-term outcome, workability, HRQoL and neuropsychological functioning in patients with encephalitis and ASM of various and unknown etiology. Our main findings were 1) encephalitis and ASM patients have low short-term HRQoL scores compared to the normal population and displayed reduced workability, with more pronounced reductions in encephalitis, 2) in encephalitis, HRQoL as reflected by MCS, correlated with a putative neurotoxic imbalance of the KP, and 3) encephalitis displayed more long-term neurocognitive deficits.

Persisting complaints after surviving encephalitis are well known, especially for HSV1-encephalitis^3,4^. Less is known on outcome and neurocognitive functioning in patients with encephalitis with non-HSV or unknown etiology and ASM. Although comparison with other studies is hampered by different test batteries and time to follow-up, our data support previous findings of reduced HRQoL for patients with ASM, at least on the short-term^8^. However, after 12 months the number of neurocognitive tests with a z-score below −1 SD in the ASM group were low (3%), indicating good recovery with no/minimal neurocognitive sequelae. For patients with encephalitis, although no deficits were found in some individuals, the higher numbers (14%) of neuropsychological tests with a z-score below −1 SD indicate more neurocognitive deficits in this group. A major finding in this study was that for patients with encephalitis, MCS (as a marker of HRQoL) was inversely correlated with neopterin levels. IFN-γ is an activator of IDO, the first step of the KP, and neopterin is thought to be a stable and reliable marker of IFN-γ activity^43,44^. The CSF level of IFN-γ at time of diagnosis has been associated with worse outcome at 3 months in patients with HSV1-encephalitis^13^. During inflammation, activation of the KP results in the formation of metabolites with potential neurotoxic (e.g. QA and 3-HK) and neuroprotective (e.g. KYNA) effects^45^. For the putatively neuroprotective/neurotoxic ratio of the KP (i.e. KYNA/(3-HK + QA)), a positive association was found. Others have found that worse outcome is associated with low KYNA levels during the acute phase, while elevated levels of KYNA has been detected more than one year after onset of HSV1 encephalitis^15^. KYNA is an antagonist of the NMDA receptor, and may antagonize the neurotoxic effect exerted by the QA. However, activation of the KP and elevated levels of KYNA have also been associated with cognitive impairment and psychotic symptoms, possibly through antagonism of the alpha- 7 nicotinic acetylcholine receptor (α7nAchR)^46^. Our findings suggest that an imbalance of the KP in the direction of neurotoxic metabolites during the acute-phase may be associated with worsened HRQoL for patients with encephalitis. This influence may, at least partly be mediated by increased IFN−γ activity reflecting increased Th1 cellular immune activity with neopterin as a reliable marker.

The strength of this study is the prospective character and the reported findings of reduced HRQoL and persisting complains for as long as two months after discharge. Especially for patients with ASM these findings are of clinical relevance. Moreover, the proportion of identified cause in patients with ASM is high (74%), and shows, as suggested by others, that extensive utilization of PCR in all patients with ASM may increase the number of patients with identified cause^47^. The study has some limitations. The relatively high proportion of patients lost to follow-up (26% and 28% in the encephalitis and the ASM group, respectively) may have biased the results. In the encephalitis group, three patients died as a consequence of their encephalitis, and for three patients comorbidity such as cancer and psychiatric disease led to exclusion from the follow-up study. Moreover, patients that were lost to follow up in the ASM group (Fig. 1) may have represented the healthiest of those diagnosed with ASM. However, except from a higher proportion of detected agents in the encephalitis group not included in the follow-up study, there were no significant differences in clinical and epidemiological characteristics between included and not included patients. Furthermore, pathogenesis and outcome of CNS infections has been shown to depend on the causing agent^48^. However, because of heterogeneous etiology and the small study groups, no sub-analyses could be done, and the statistical power of the analyses is low. We suggest that the reduced HRQoL and neurocognitive functioning in the encephalitis group is caused by the parenchymal character of the infection, including KP activation, but older age and male dominance in the encephalitis group might cause a bias. Likewise, the influence of CNS infection on HRQoL may be overestimated, especially for the ASM group, which consisted of many females in their thirties with a busy family life. Lastly, correlation does not mean causality and given the limited sample size and the explorative nature of the study, there is a need for larger studies to evaluate these issues.

In conclusion, encephalitis, but also ASM have substantial short-term influence on HRQoL and workability. However, according to this study, the long-term prognosis for ASM patients seems good. Our study suggests a link between impaired HRQoL and metabolites of the KP in patients with encephalitis that might represent a novel target for therapy. This should be further evaluated in larger encephalitis cohorts with CSF analyses during follow-up.

## Data Availability

The datasets analyzed during the current study are available from the corresponding author on reasonable request.
